# Measurements of 17β-estradiol levels in mice for migraine research

**DOI:** 10.1186/1129-2377-14-S1-P92

**Published:** 2013-02-21

**Authors:** K Chan, S Labruijere, I Garrelds, A Danser, C Villalón, A MaassenVanDenBrink

**Affiliations:** 1 Erasmus Medical Center, Netherlands; 2 Cinvestav Coapa, Czda, Mexico

## Introduction

Since migraine prevalence is 2-3 times higher in women than in men, especially during the reproductive years, fluctuations in female sex hormone levels seem to be one of the key factors involved in the pathogenesis of migraine. During the last decade, lots of animal research in migraine has been performed on mice, since this species is well suited to create transgenic animal models1. To investigate the effect of female sex hormones in a murine model, it is important to analyse the hormone levels and/or to determine the hormone cycle in mice. Although no reliable assay has been available to measure murine plasma estrogen, recently 17β-estradiol assays have been suggested to be able to quantify these hormone levels.

## Purpose

We set up a pilot study to test 3 different ELISA kits described in the literature.

## Methods

Plasma samples and vaginal smears of female mice (10-11 weeks) were collected at two different time points: 3 days before and 2 weeks after ovariectomy (OVX), when the animals were sacrificed. Weights of uterus were also collected. Blood samples were tested in 3 different ELISA kits obtained from Cayman Chemical (Ann Arbor, MI, USA), GenWay Biotech (San Diego, CA, USA) and Calbiotech (Spring Valley, CA, USA), respectively.

## Results

All the tested ELISA assays did not show any differences in 17β-estradiol levels before and after OVX. Likewise, no differences in 17ß-estradiol levels between sham-operated and OVX animals were observed using these assays. Data from vaginal smears and uterus weights (sham: 42.7-94.8 mg, OVX: 16.2-52.0 mg), however, confirm that OVX was successfully performed.

## Conclusion

We conclude that the tested ELISA assays are not capable of precisely determining 17β-estradiol levels in mice. Since vaginal smears, uterus weights and ovary staining are indicative of the phase of the cycle, in future studies these parameters may be used to analyze the hormonal status in mice.
